# Using compensating variation to measure the costs of child disability in the UK

**DOI:** 10.1007/s10198-017-0893-7

**Published:** 2017-04-13

**Authors:** Mariya Melnychuk, Francesca Solmi, Stephen Morris

**Affiliations:** 1 Department of Applied Health Research, University College London, Room 112, 1-19 Torrington Place, London, WC1E 7HB UK; 2 Division of Psychiatry, Faculty of Brain Sciences, University College London, London, UK

**Keywords:** Compensating variation, Child disability, Matching, Living standards, C81, D1, I1, J1

## Abstract

The objective of disability policy is to create a society where people with disabilities and their families enjoy an equal standard of living to those without disabilities, though evidence to underpin policy is sparse. We defined the compensating variation (CV) of child disability as the amount of additional income a family with a disabled child would require to achieve the same living standards as a similar family without a disabled child. The aims of this study were to estimate the CV for child disability and to explore how this varied for different levels of disability and reference levels of living standards. Using data on 54,641 families from the Family Resources Survey (2004–2012), we matched families with (cases) and without (controls) a disabled child on family and child characteristics plus living standards and calculated the income difference inclusive of disability benefits. Our findings suggest that across families with the most disabled children, a compensating variation equal to an extra £56–£79 a week was required to achieve the same living standards as matched families without a disabled child compared with the mean level of state disability benefit £47–£71 a week in this group.

**Electronic supplementary material:**

The online version of this article (doi:10.1007/s10198-017-0893-7) contains supplementary material, which is available to authorized users.

## Introduction

In 2011/12 around 800,000 children aged 0–15 years lived with a disability in Great Britain [10]. Evidence suggests that families with a disabled child have lower income, living standards and levels of social inclusion [10] and that living with a disabled child is associated with parental unemployment [20] and family break-up [6]. UK data show that 21% of children with a disability live in poverty compared to 16% of children without a disability [10].

Childhood experience of socio-economic disadvantage has been shown to be associated with long-term adverse physical and mental health outcomes [8, 28] possibly via the persistence of a lower socio-economic status in adulthood [21]. In turn, evidence suggests that childhood disability and poor health can lead to lower long-term quality of life [30] and socio-economic status in adulthood [33]. Therefore, the double burden of disability and economic disadvantage could compromise a child’s health and ability to thrive throughout their life.

In the UK, families of children under 16 years of age with a disability causing difficulties with walking or in need of extra care are entitled to a weekly Disability Living Allowance (DLA) ranging from £21.80 to £139^1^ (2014 prices) depending on the level of need. To qualify for DLA the child has to either ‘need more looking after than a child of the same age who does not have a disability’ or have difficulties ‘getting about’, or both. The amount of DLA the child is eligible to receive is therefore calculated on the basis of both a care (i.e. the level of looking after they need) and a mobility (i.e. the level of help they need getting about) component. For the care component the child can receive a low rate (£21.80 a week) if they need help for some of the day or night, a middle rate (£55.10) if they need frequent supervision during the day or night, or a high rate (£82.30) if they need constant help during both day and night or if they are terminally ill. For the mobility component, the child is eligible to receive a low rate (£21.80) if they can walk but need help and/or supervision when outdoors or a high rate (£57.45) if they are unable to walk, or to walk long distances, if their health could be affected if they tried to walk, or if they’re blind or severely sight impaired (“Disability Living Allowance (DLA) for children” [12]).

We analyse the costs borne directly by the families with disabled children rather than aiming to identify and measure all the costs related to child disability, including the direct, indirect and intangible dimension borne by the public sector [5, 29]. There are four methodological approaches that have been used to calculate the extra costs of disability: the subjective approach, the comparative approach, the budget standard approach and the standard of living approach. Using the subjective approach [22], The Disablement Income Group study [35] and Woolley [37] asked disabled people about their additional expenditures and estimated the extra costs of disability. Studies using the comparative approach [19, 23] collect data on actual expenditures from both disabled and non-disabled people to compare spending patterns and show where priorities differ. Both approaches may underestimate the costs of disability because responses will be affected by the budgetary constraints of respondents. For example, if families affected by disability have lower incomes than families not affected by disabilities, they may spend less even though their needs may be greater. Hence observed spending patterns may not reflect the true costs of disability. To tackle this limitation, the Centre for Research in Social Policy (CRSP) developed the budget standard approach in which respondents are asked to provide a list of items required for a *reasonable* standard of living [13, 34]. Responses are obtained from respondents affected by disability; the items are then individually costed and summed to estimate the total spending requirements of those affected by disability. Limitations of this approach are that it is based on stated rather than revealed preferences and that it does not measure ‘extra’ cost associated with disability as it does not compare spending requirements of disabled and non-disabled people. The standard of living approach was introduced by Berthoud et al. [2] and then used by Zaidi and Burchardt [38]. It relies on the assumption that disabled people experience a lower standard of living compared with non-disabled people with the same income because of spending money on goods and services associated with their disability. Respondents are first ranked using an index of living standards derived from items unrelated to their disability. For each standard of living, it is then possible to calculate the difference in income between disabled and non-disabled respondents. This difference can be conceptualised as the extra income that a disabled individual requires to achieve the same living standards of a non-disabled individual. This approach has been used to calculate the costs of disability among adults [38], but this approach has not been used to estimate the costs of childhood disability.

Little evidence exists quantifying the costs of child disability. Dobson and Middleton [13] used the budget standard approach and compared the minimum essential budgets for disabled children and those for children without a disability and calculated that it costs on average £99 a week (1997 prices) to bring up a child with a severe disability from birth to 17 years of age.^2^ They calculated that disability benefit would need to increase by £30–£80 per week in order to meet the minimum essential needs. Dobson et al. [14] suggested the situation has improved since 1997 and the difference between the maximum benefit income and the essential costs was £28 a week (2000 prices). A limitation in both studies was that the budgets were developed for very precise definitions of disability, limiting generalisability to other forms of disability.

In this study we use the living standard approach to estimate the costs of child disability. We estimate the amount of extra income required by families with a disabled child compared to families without a disabled child to achieve the same living standards.

## Theoretical background

**Fig. 1:**
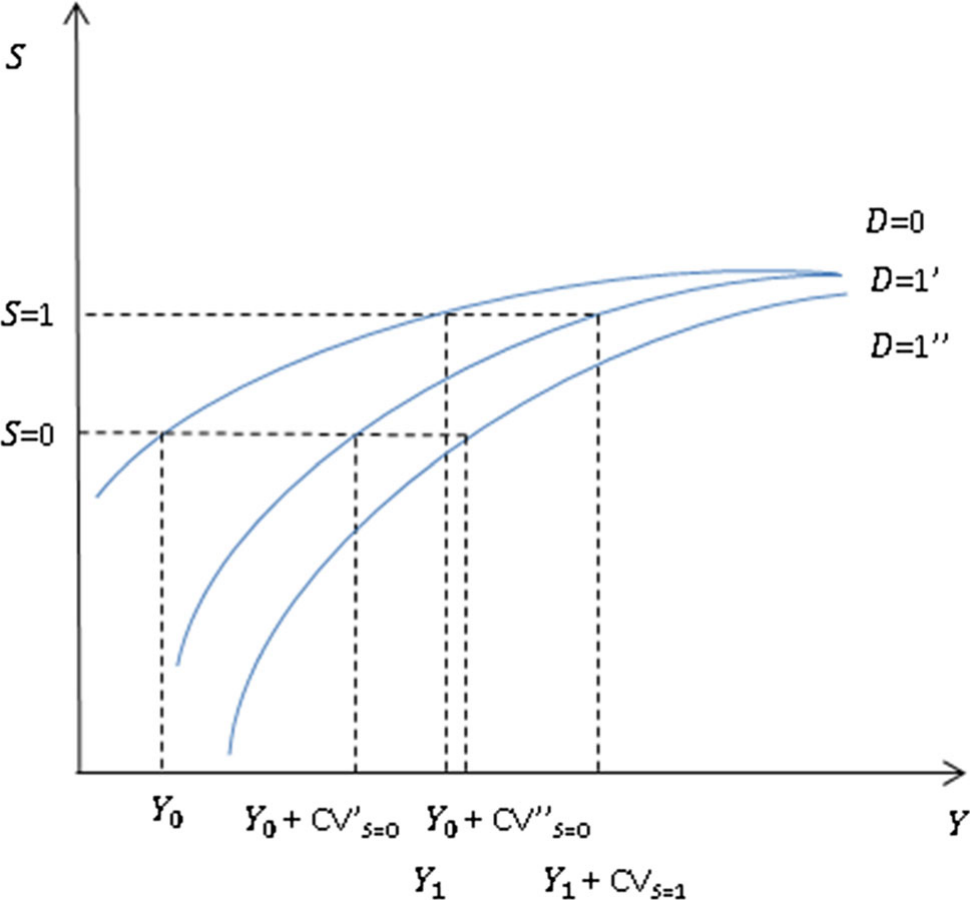
Relationship between income, standard of living and disability

Hancock et al. [17] used the concept of compensating variation (CV) to estimate the costs of disability in adults. In the case of adults, the CV is the additional income that a disabled adult needs to achieve the same living standards as a similar adult who is not disabled. In the case of childhood disability, we define the CV as the additional income that a family with a disabled child needs to meet the same living standards of a family whose child is not disabled.^3^ Unlike in the adult case, which focuses on individuals, we focus on families because children are not independent. Graphically, the CV can be illustrated by plotting the curve relating family income (*Y*, which includes all income likely to affect living at standards including disability benefits or subsidised care services) and standards of living (*S*).^4^ We hypothesise the curve is upward sloping from left to right, with a diminishing impact of additional income (Fig. 1). Curves are plotted for a family with (*D* = 1) and without (*D* = 0) a disabled child. In the figure we assume the curve for a family with a disabled child lies below the curve for a family without a disabled child, on the assumption that if the two families received the same income the one with the disabled child would have lower living standards. We also assume the curves eventually coincide at high levels of income. For a given level of *S* it is possible to calculate the difference in *Y* between the two families. For instance, given the curves *D* = 0 and *D* = 1′ to achieve *S* = 0 a family with a disabled child needs an income equal to *Y*_*0*_ + CV’_*S*=0_ compared with the family without a disabled child, which needs *Y*_*0*_. Hence in this situation, for a reference level of living standards given by *S* = 0 the CV is CV′_*S*=0_. The CV is likely to vary by the extent of disability (affecting the shape and positioning of the curve *D* = 1). For example, compared with *D* = 1′, a family with a child with a higher level of disability might have a curve given by *D* = 1″, and a CV given by *Y*_*0*_ + CV″_*S*=0_, where CV″_*S*=0_ > CV′_*S*=0_. At a different reference level of living standards *S* the CV also may change. For example, at *S* = 1 the CV is given by CV′_*S*=1_, where based on the assumptions made CV′_*S*=1_ < CV′_*S*=0_.

In the light of the above, the aim of this study is to estimate the CV for child disability and to explore how this varies for different levels of disability and reference levels of living standards.

## Data and variables

### Data

The Family Resources Survey (FRS) is a large repeated cross-sectional survey sponsored by the Department of Work and Pensions (DWP), which was started in 1992 covering Great Britain and extended in 2002/03 to cover the whole of the UK. Broadly, the FRS aims to provide detailed information on the incomes and circumstances of private households in the UK. The survey also collects information on difficulties due to ill health or disability and asks a series of questions aimed at measuring material deprivation. The sample size is approximately 25,000 households each year, drawn using a stratified cluster probability sample from the Royal Mail’s small users Postcode Address File (PAF), which ensures the data are nationally representative [15].

In this study, we employed data covering eight rounds of data collection (from 2004–2005 to 2011–2012), as these contained all the necessary data for our analyses. Our sample included all children aged 0–15 years with complete information on all variables employed in the analyses. We excluded children aged 16–18 years since Disability Living Allowance (DLA) arrangements are different in this age group (i.e. the benefits are given directly to the child).

We estimated the child disability index at the child level; however we refer to the ‘benefit unit’ (i.e. family) as our main unit of analysis, as data on family type and characteristic, and lack of essential items is recorded at that level. A benefit unit is an adult, their partner (if applicable) and any dependent children they are living with. We limited our analysis to families with either no or one disabled child; we did not include families with more than one disabled child because of difficulties separating the effects of multiple disabled children in the same family (*N* = 1663, 2.0%). We included families with disabled adults, including whether or not there was an adult with a disability in the matching process.

### Child disability

We developed a measure of child disability that reflects the definition of disability included in the Disability Discrimination Act (DDA), 1995 and 2005, i.e. a child with a longstanding illness or disability that substantially impacts on their day-to-day activities. This definition attempts to exclude children with short-term conditions or those with conditions with no impact on day-to-day activities [3, 24].

For each child, families were asked if they had a longstanding illness, disability or infirmity. Following a positive answer families were asked to record up to ten areas of the child’s life that were affected by this problem or disability. Possible answers were: (1) moving, (2) lifting, (3) manual dexterity, (4) continence (bladder control), (5) communication (speech, hearing or eyesight), (6) memory and learning, (7) recognising when in physical danger, (8) physical co-ordination and (9) other; additionally, families could also answer that (10) none of these areas were affected by disability. We defined a disabled child as any child whose parent had answered yes to the main question and had additionally answered yes to any of questions (1)– (9). Children whose families had answered ‘none’ at the follow-up question are excluded from the analysis because details and extent of the disability were unclear. All other children were considered non-disabled.

We created a disability index that could be used to stratify the sample of disabled children into sub-groups. To do this we estimated relative weights for each of the nine areas of the child’s life affected by disability (similar to the one used by [17]). We estimated the following logit model:

1 $$ \begin{aligned} Y_{i} = \mu_{0} + \alpha_{1} {\text{mobility}}_{i} + \alpha_{2} {\text{lifting}}_{i} + \alpha_{3} {\text{dexterity}}_{i} + \alpha_{4} {\text{continence}}_{i} \hfill \\ + \alpha_{5} {\text{communication}}_{i} + \alpha_{6} {\text{memory}}_{i} + \alpha_{7} {\text{danger}}_{i} { + }\alpha_{8} {\text{coordination}}_{i} \hfill \\ + \alpha_{9} {\text{other}}_{i} + \gamma X^{{\prime }} + \varepsilon_{i} \hfill \\ \end{aligned} $$

where *Y*_*i*_ is a binary variable delineating whether or not the child was receiving disability benefits, $\mu_{0}$ is a constant term, $\alpha_{i}$ are the coefficients of nine indicators of disability, *γ* is a coefficient on other explanatory covariates *X′* that may influence the likelihood of receiving disability benefits (summarised in Appendix Table A1), and $\varepsilon_{i}$ is an error term.

We used the results of the logistic regression to create a disability index value for each disabled child in the sample as a function of the nine indicators of disability. The index was the linear index from the logistic regression model—the weighted sum of the indicators using the estimated logit coefficients $(\hat{\alpha }_{i} )$ as weights. The weights were normalised to produce a range between 0 and 1 by calculating the proportion of each $\hat{\alpha }_{i}$ in the total sum of coefficients $\sum\nolimits_{i = 1}^{9} {\hat{\alpha }_{i} }$. We created another version of the index where instead of using whether or not the child received any disability-tested public support as the dependent variable in the regression we used whether or not the child was registered as disabled with the LA. The two versions of the disability index were highly correlated (*r* = 0.98) and we present results for the receipt of disability benefits measure.

We divided families with a disabled child into quartiles using disability index scores, with quartile 1 reflecting the lowest level of disability and quartile 4 the highest level. Each quartile contained approximately 25% of disabled families (the proportions were only approximate because of tied values).

### Living standards

The FRS comprises a set of questions aimed at capturing material deprivation at the benefit unit level. These questions ask the head of the family whether the latter: can afford and has; would like to have, but cannot afford; or can afford but does not want a number of goods perceived as necessities by families with (11 questions) and without children (10 questions) [25].^5^ Of the 21 questions included in the FRS we employed 12 questions that were asked to the whole sample at each survey wave (2004/05–2011/12) to derive our living standards index (Appendix Table 10).

Previous studies have suggested different ways of developing a living standard index using similar data [17, 36]. We created an index that accounted for whether families could afford items that they consider desirable, using a form of prevalence weighting, with weights reflecting the relative necessity of owning an item within our sample [16]. For instance, fewer people might be able to afford a holiday than a pair of winter shoes, but, if winter shoes are considered by a greater proportion of participants as a desirable item, then not being able to afford it would give this item a greater weight in our living standards index. We therefore calculated the living standards index in three steps. First, we gave a score of 1 (score of 0) to participants reporting being able (not being able) to afford an item, regardless of whether they owned it.^6^ Second, we multiplied this binary variable by the proportion of people who considered the item desirable (i.e. those who owned it, plus those who wanted it but could not afford it). Finally, we calculated the living standards index (LSI) as follows:

2 $$ {\text{LSI}} = \frac{{\mathop \sum \nolimits_{i = 1}^{M} x_{i} w_{i} }}{{\mathop \sum \nolimits_{i = 1}^{M} w_{i} }} $$

where *x*_*i*_ represents whether a family can (i.e. the family has or does not have because the item is not important, but they could afford it) or cannot afford an item, and *w*_*i*_ represents the proportion of the sample who regarded the item as desirable (i.e. those who have the item and those who would like to have it, but cannot afford it). Since 10% of the families had missing data for one or more questions needed to derive the living standard index, using this procedure, we were able to scale the index according to the total possible score each family could have obtained if they could have afforded each item, regardless of the number of missing answers to the living standards questions, i.e. 1 ≤ *M* ≤ 12. As a sensitivity analysis we used the same procedure but employed weights reflecting the proportion of the sample that could afford each item [11]. The two indices were highly correlated (*r* = 0.99) and we present results for the first measure, based on whether each item was considered to be a necessity.

We divided families into two groups based on their LSI value, those with a score of 1 (families with the highest living standards who can afford all the items they regard as desirable) and those with a score <1 (who could not afford at least one of the items they regard as desirable).

### Income measure

To measure family income we include all available economic resources that determine living standards of the family [26, 38]. We consider three components [17]. First, income from all sources, including earnings, self-employment, investments and pensions, inflated to 2011/12 prices. Each of these components can be measured as both the gross and net of taxes, the latter reflecting disposable income; this is the measure we use here. Second, all types of benefits, including disability benefits, also inflated to 2011/12 prices. Inclusion of benefits is important because they also contribute to the observed family living standards, i.e. we cannot observe the level of living standards in the absence of these benefits. Third is the value of non-medical formal care services.^7^ The FRS records whether or not the child received social care provided by the LA (e.g. home help or home care worker) and/or home nursing care, including data on the total amount of hours provided of both types of care per week. We cost each hour of social care at £23.45 and each hour of home nursing care at £68.0 (2011/12 prices) [9]. We then calculated the average cost of formal care based on the mean number of hours of care received per week multiplied by the unit cost for both types of care.

It is unclear whether income should be measured as the net of housing costs [38]. For instance, housing quality is a consumption choice for relatively wealthy families, suggesting income inclusive of housing costs should be preferred. On the other hand, for families receiving housing benefits or tenants in social housing, an increase in rent raises their before-housing-cost income (because housing benefits increase with rents) without providing any additional disposable income. Depending on the extent to which the housing costs are believed to be at the discretion of the family or considered as a fixed cost, one may wish to subtract them from the net income measure.

Our main income measure is net income from all sources inclusive of benefits. A second income measure, ‘discretionary income’, is net income with benefits excluding housing costs (water and sewerage rates, rent, mortgage interest, insurance and service charges). We also include both measures with and without the estimated value of formal care. To adjust income for household composition, we include variables for the numbers of adults and children living in each household plus ages of children in the matching process [26, 38].^8^

### Other variables

In the matching process we included a number of variables describing socio-demographic and socio-economic characteristics of the children and their families. Child characteristics were age and gender. Family characteristics were: the number of dependent children (linear term, 1–8); a binary variable indicating the presence of a disabled adult in the benefit unit (yes/no); a binary variable describing the type of the benefit unit (main respondent single/living as a couple); years of schooling of the household reference person after compulsory education (a linear term where reflecting each additional year spent in education over the age of 16 years), a categorical variable (five categories) for total savings to control for family wealth and a categorical variable for employment status (broad ILO definition, three categories). We also used four geographical indicators for the grouped Government Office Region (London; South East; rest of England; Northern Ireland, Wales and Scotland) and eight indicators for the FRS round of data collection (year).

## Statistical methods

### Matching technique

Non parametric methods using propensity score matching can offer a more appropriate approach compared with parametric methods for estimating CVs because they attempt to simulate a randomised setting using observational data. It has been shown that the parametric method for estimating CVs provides unstable results and can overestimate the cost of disability [17]. Following Rosenbaum and Rubin [31] and Heckman et al. [18], we employed propensity score matching to match families with and without a disabled child in an attempt to account for potential bias in estimating the impact of child disability on family income. Let *Y*_1_ be family income when a family has a disabled child (*D* = 1) and *Y*_0_ family income when a family does not have a disabled child (*D* = 0). The observed income *Y* is:

3 $$ Y = DY_{ 1} + \left( { 1 - D} \right)Y_{0} $$

so that when *D* = 1 we observe *Y*_1_ and when *D* = 0 we observe *Y*_0_. We wish to estimate the average treatment effect on the treated (*ATT*) of child disability on income for the families with a disabled child (the ‘treated’ group), defined as:

4 $$ {\text{ATT }} = {\text{E}}[Y_{ 1} - Y_{0} |D = 1] = {\text{E}}[Y_{ 1} |D = 1\left] { - {\text{E}}} \right[Y_{0} |D = 1] $$

However, we cannot observe simultaneously both E (*Y*_1_|*D* = 1) and E (*Y*_0_|*D* = 1), i.e. we cannot observe what the income of families with a disabled child would be if the child was not disabled. However, we do observe the income of families without a disabled child. We define a propensity score as the conditional probability of having a disabled child, given family and child observed characteristics *X*:

5 $$ {\text{p}}\left( D \right) = { \Pr }(D = 1|X) = {\text{E}}\left( {D|X} \right) $$

Based on Rosenbaum and Rubin [31], if having a disabled child is random conditional on elements of *X* it is also random conditional on p (*X*), and we can use this to match families with a disabled child to families without a disabled child and estimate the income difference:

6 $$ ATT = {\text{E}}[Y_{ 1} - Y_{0} |D = 1] = {\text{E}}[Y_{ 1} - Y_{0} |D = 1,{\text{ p}}\left( X \right)] = {\text{E}}[Y_{ 1} |D = 1,{\text{ p}}\left( X \right)\left] { - {\text{E}}} \right[Y_{0} |D = 0,{\text{ p}}\left( X \right)] $$

More specifically, the *ATT* is the difference in income between the families with a disabled child and matched families without a disabled child. Formally, in order to derive (6) given (5), we need to demonstrate that families with a disabled child and matched families without a disabled child are on average observationally identical. In other words, families with the same propensity score should have the same distribution of observable characteristics, regardless of having a disabled child.

Propensity scores can be used to create matched observations with similar distributions of the covariates *X*, but do not require exact matching on all of the individual components of *X*. The CV is the additional income that a family with a disabled child needs to meet the same living standards of a family whose child is not disabled. One option is to include living standard *L* in the observed characteristics *X*, but the matching process might not lead to exact matching on *L*. An alternative approach, adopted here, is to make *L* external to *X* and match families according to both *L* and p(*X*):

7 $$ {\text{ATT }} = {\text{E}}[Y_{ 1} - Y_{0} |D = 1] = {\text{E}}[Y_{ 1} - Y_{0} |D = 1,L,{\text{p}}\left( X \right)] = {\text{E}}[Y_{ 1} |D = 1,L,{\text{p}}\left( X \right)\left] { - {\text{E}}} \right[Y_{0} |D = 0,L,{\text{p}}\left( X \right)] $$

### Compensation variation

The propensity score in (7) is computed from a univariate probit model in which the units of analysis are families identified by whether or not they have a disabled child (disability index >0), their living standards and values of the covariates *X*. In the first analysis we regressed whether or not the family had a disabled child (1 = yes, 0 otherwise) against the covariates *X*. The propensity score was calculated as the predicted probability from this model. Then, for each family with a disabled child we selected a match from the pool of families without a disabled child with the same value of living standards (based on the first four digits of the index) and the closest propensity score within the common support area. Common support (i.e. calliper size) was defined to be within one quarter of the standard deviation range of the estimated propensity score [7, 32].^9^ We performed one-to-one nearest-neighbour Mahalanobis matching within the calliper with replacement. For every matched pair we calculated the CV in (7) and the associated standard error using the method proposed by Abadie and Imbens [1].

We ran three sub-group analyses. First, we reran our analyses for sub-groups defined by the quartiles of disability. We created four data sets, each containing all the families with no disabled children plus families with a disabled child in one quartile of disability. We then matched families and calculated the CV for each quartile of disability using the same approach described above. Second, we stratified by the two living standards groups (LSI < 1, LSI = 1) across all levels of disability combined. Third, we stratified our analyses by both quartiles of disability and living standards groups.

## Results

### Sample characteristics

The sample comprised 85,627 children from 52,556 families. A total of 4320 (5%) children had a longstanding illness, disability or infirmity in at least one of the nine areas used to create the disability index. The majority of children lived in a household with two adults with no disabilities and, on average, two children (Table 1). A higher proportion of children with at least one disability were older, male, lived in a single parent household with fewer siblings, lived with an adult who also had a disability and fewer years of schooling, and had fewer family savings. Note that for the analyses using discretionary income, we excluded 7883 (9%) children from families with missing data on housing costs, giving a sample of 77,794 children from 47,995 families; the above trends were also found in this sample.

**Table 1:** Sample characteristics

	All, *N* (%)	Child disability		*p* value
		No, *N* (%)	Yes, *N* (%)	
Total	85,627 (100%)	81,307 (94.95%)	4320 (5.05%)	
Gender				
Male	43,711 (51.05%)	40,970 (50.39%)	2741 (63.45%)	<0.0001
Female	41,916 (48.95%)	40,337 (49.61%)	1579 (36.55%)	
Government region				
London	8743 (10.21%)	8365 (10.29%)	378 (8.75%)	0.001
South East	9996 (11.67%)	9498 (11.68%)	498 (11.53%)	
Wales, Scotland, Northern Ireland	23,566 (27.52%)	22,420 (27.57%)	1146 (26.53%)	
Rest of England	43,332 (50.59%)	41,024 (50.36%)	2298 (53.19%)	
Type of BU				
Single	21,586 (25.21%)	19,934 (24.52%)	1652 (38.24%)	<0.0001
Couple	64,041 (74.79%)	61,373 (75.48%)	2668 (61.76%)	
Adult with disability in BU				
No	72,040 (84.13%)	69.231 (85.15%)	2809 (65.02%)	<0.0001
Yes (at least one parent)	13,587 (15.87%)	12,076 (14.85%)	1511 (34.98%)	
Year				
2004/05	12,822 (14.97%)	12,232 (15.04%)	590 (13.66%)	0.274
2005/06	11,640 (13.59%)	11,044 (13.58%)	596 (13.80%)	
2006/07	11,150 (13.02%)	10,603 (13.04%)	547 (12.66%)	
2007/08	10,411 (12.16%)	9857 (12.12%)	554 (12.82%)	
2008/09	10,414 (12.16%)	9888 (12.16%)	526 (12.18%)	
2009/10	10,279 (12.00%)	9744 (11.98%)	535 (12.38%)	
2010/11	10,353 (12.09%)	9829 (12.09%)	524 (12.13%)	
2011/12	8558 (9.99%)	8110 (9.97%)	448 (10.37	
Total savings				
No savings	4,116 (4.81%)	3857 (4.74%)	259 (6.00%)	<0.0001
Savings less than £1500	46,241 (54%)	43,428 (53.41%)	2813 (65.12%)	
Savings over £1500 and up to £20,000	22,001 (25.69%)	21,198 (26.07%)	803 (18.59%)	
Savings over £20,000	10,609 (12.39%)	10,240 (12.59%)	369 (8.54%)	
Did not want to say	2660 (3.11%)	2584 (3.18%)	76 (1.76%)	
Employment status				
In employment	60,835 (71.05%)	58,424 (71.86%)	2411 (55.81%)	<0.0001
ILO unemployed	3038 (3.55%)	2849 (3.50%)	189 (4.38%)	
Economically inactive	21,754 (25.41%)	20,034 (24.64%)	1720 (39.81%)	
Sex of household reference person				
Male	52,503 (61.32%)	50,410 (62.00%)	2093 (48.45%)	<0.0001
Female	33,124 (38.68%)	30,897 (38.00%)	2227 (51.55%)	

	Mean (SD)	No, mean (SD)	Yes, mean (SD)	*p* value
Child age	7.42 (4.67)	7.34 (4.69)	8.96 (4.15)	<0.0001
Age household reference person left full time education (years above 16)	1.08 (2.15)	1.10 (2.17)	0.67 (1.77)	<0.0001
Number of dependent children in household	2.19 (0.99)	2.19 (0.99)	2.11 (0.99)	<0.0001

### Disability index

**Fig. 2:**
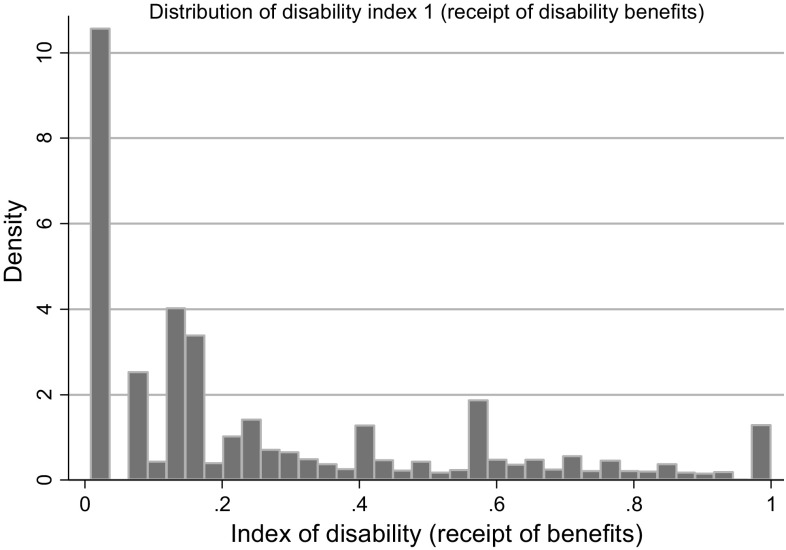
Distribution of index of disability (for disabled children only)

Results of the logit model used to create the disability index are in Table 2.^10^ The normalised weights derived from the coefficients produced an index in which communication problems and appreciation of danger are heavily weighted, reflecting the importance assigned to these dimensions of disability by the public support for disability and participants own perceptions of need. The distribution of values in the resulting index (only for children with disabilities) is plotted in Fig. 2, showing a large grouping of children with a low level of disability according to the index and declining numbers at higher levels of the disability index.

**Table 2:** Logit coefficients and normalised weights used to compute disability index

Area affected	Logit coefficients, (95% CI)	Normalised weights
Mobility	0.427 (0.23; 0.62)	0.135
Lifting	0.226 (−0.03; 0.48)	0.072
Dexterity	0.214 (−0.02; 0.45)	0.068
Incontinence	0.391 (0.19; 0.60)	0.124
Communication	0.496 (0.31; 0.68)	0.157
Memory	0.231 (0.02; 0.44)	0.073
Appreciation of danger	1.045 (0.84; 1.25)	0.331
Co-ordination	0.034 (−0.17; 0.24)	0.011
Other	0.089 (−0.08; 0.25)	0.028
Sum	3.154	1.0000

### Living standards index

**Fig. 3:**
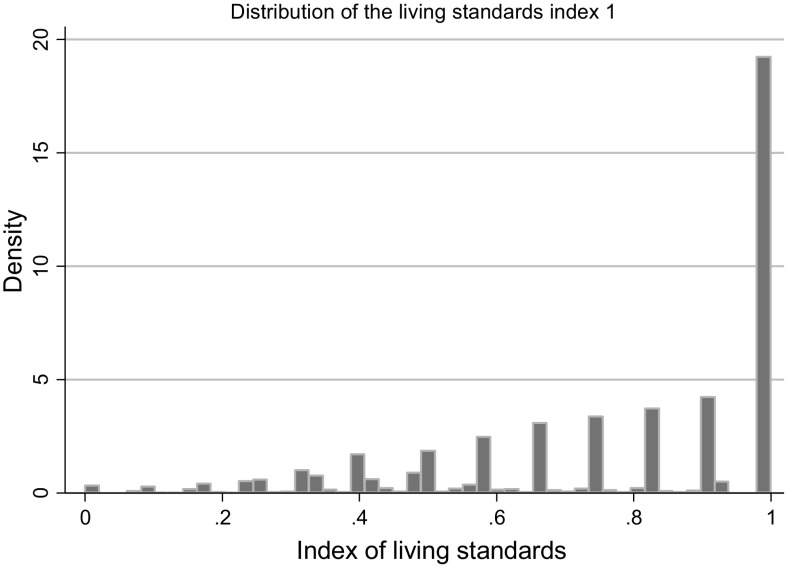
Distribution of LSI (for all families)

The distribution of values in the resulting index (for all families) is plotted in Fig. 3, showing a grouping of children with relatively low levels of disability according to the index, and declining numbers at higher levels of disability. Approximately 40% of the sample had an LSI value equal to one, indicating families that could afford all of the items in the index.

### Income

Mean (SD) net household income per disabled child per week was £608.73 (£481.38) and per child with no disability it was £715.18 (£671.79) (Table 3). Mean (SD) net income increased with level of disability ranging from £583.16 (£369.11) to £650.08 (£458.87). When using discretionary income, mean income for families with a disabled child was £520.49 (£483.10) and for non-disabled children it was £617.80 (£670.49). Adding the value of formal care to net and discretionary income did not change the values for families without a disabled child, but on average across all disabled children increased income by approximately £7. Increasing income with severity of disability is observed for all four measures of income employed, and the addition of formal care has a larger effect on income of families with more severely disabled children (adding nothing to household income among the least disabled children, to £20 with the most severely disabled children).

**Table 3:** Mean income by level of disability and income measure

Disability	Measure of income (mean (SD) [*s*])			
	Net income	Net income + formal care	Discretionary income	Discretionary income + formal care
No disability	715.18 (671.79) [81,307]	715.18 (671.79) [81,307]	617.80 (670.49) [73,798]	617.80 (670.49) [73,798]
All disabled	608.73 (481.38) [4320]	615.63 (494.39) [4,320]	520.49 (483.10) [3996]	527.42 (496.16) [3996]
Quartile 1	583.16 (369.11) [1247]	583.65 (369.61) [1247]	494.04 (359.20) [1167]	494.56 (359.74) [1167]
Quartile 2	596.72 (585.88) [1173]	598.30 (586.74) [1173]	511.50 (594.29) [1083]	513.21 (595.31) [1083]
Quartile 3	610.20 (492.73) [817]	614.95 (499.41) [817]	521.99 (501.39) [745]	526.80 (508.06) [745]
Quartile 4	650.08 (458.87) [1083]	671.74 (502.69) [1083]	559.94 (458.61} [1001]	581.60 (502.67} [1001]

Household income is also found to vary by living standards (Table 4). In every case income was higher in families with LSI = 1 compared with those with LSI < 1. In families with LSI = 1, across all four income measures, income was higher in families without a disabled child, and there was little variation in income by severity of disability. In families with LSI < 1, for every income measure, income increased with severity of disability, and in disability quartiles 1–3 the values were lower than those for families without a disabled child, whereas in the most severely disabled quartile income was higher. The addition of formal care costs had a larger effect on families with LSI < 1 compared to those with LSI = 1.

**Table 4:** Mean income by level of disability, living standards and income measure

Disability	Measure of income (mean income difference (SD) [N])							
	Net income		Net income + formal care		Discretionary income		Discretionary income + formal care	
	LSI < 1	LSI = 1	LSI < 1	LSI = 1	LSI < 1	LSI = 1	LSI < 1	LSI = 1
No disability	531.25 (329.38) [48,833]	991.76 (916.22) [32,474]	531.25 (329.38) [48,833]	991.76 (916.22) [32,474]	436.49 (322.27) [44,503]	893.23 (921.38) [29,295]	436.49 (322.27) [44,503]	893.23 (921.38) [29,295]
All disabled	502.52 (250.60) [3167]	900.46 (761.56) [1153]	510.24 (282.70) [3167]	905.11 (763.10) [1153]	413.91 (248.94) [2925]	811.54 (765.64) [1071]	421.65 (281.62) [2925]	816.29 (767.00) [1071]
Quartile 1	471.52 (242.09) [923]	901.20 (470.32) [324]	472.01 (242.39) [923]	901.72 (471.37) [324]	385.31 (239.37) [863]	802.70 (451.94) [304]	385.82 (239.74) [863]	803.28 (451.99) [304]
Quartile 2	477.76 (238.80) [857]	919.34 (989.56) [316]	478.82 (239.70) [857]	922.31 (990.26) [316]	389.98 (236.55) [784]	830.15 (997.3) [299]	391.14 (237.53) [784]	833.28 (998.26) [299]
Quartile 3	507.88 (221.88) [603]	898.51 (823.28) [214]	513.45 (242.68) [603]	900.94 (823.22) [214]	416.16 (224.49) [554]	828.94 (842.82) [191]	421.69 (245.19) [554]	831.66 (842.70) [191]
Quartile 4	561.97 (281.71) [784]	881.13 (694.28) [299]	587.13 (370.35) [784]	893.61 (699.22) [299]	472.23 (279.58) [724]	789.19 (695.95) [277]	497.39 (369.96) [724]	801.70 (699.81) [277]

### Compensating variation

In all of our models, the treated (disabled) and untreated (not disabled) groups were well balanced after matching on all variables employed in the estimation of the propensity scores.^11^

Based on net household income, the estimate of mean CV across all families with a disabled child was not different from zero (Table 5). Disability costs were not significantly different from zero for the least disabled children (quartiles 1–3), but £57 in the most severely disabled. Similar trends were found for the other income measures, though disability costs were higher for children in disability quartile 4 for measures including formal care (£78–£79 a week).

**Table 5:** Compensating variation by level of disability and income measure

Disability	Measure of income (mean income difference (95% CI) [*N*])			
	Net income	Net income + formal care	Discretionary income	Discretionary income + formal care
All	11.18 (−9.20; 31.56) [3918]	17.73 (−2.89; 38.35) [3918]	7.28 (−11.21; 25.76) [3603]	13.87 (−7.87; 35.61) [3603]
Quartile 1	15.19 (−14.75; 45.13) [1110]	15.62 (−14.35; 45.59) [1110]	12.86 (−17.05; 42.76) [1033]	13.31 (−16.62; 43.25) [1033]
Quartile 2	−1.16 (−44.68; 42.36) [1026]	0.48 (−43.12; 44.09) [1026]	1.87 (−43.16; 46.89) [945]	3.65 (−41.47; 48.78) [945]
Quartile 3	26.96 (−19.07; 73.00) [705]	31.09 (−15.27; 77.46) [705]	31.02 (−15.58; 78.01) [635]	35.38 (−11.96; 82.72) [635]
Quartile 4	**56.80 (17.01; 96.59) [914]**	**78.83 (37.24; 120.42) [914]**	**55.75 (15.16; 96.35) [836]**	**77.71 (35.27; 120.16) [836]**

Figures in bold are significantly different from zero at the 5% level

When disaggregating families by livings standards, in the higher living standards group (LSI = 1) the CV was not significantly different to zero with any income measure and at all levels of disability (Table 6). Pooling across all families with a disabled child with LSI < 1, the CV was significantly different to zero in all four income measures, ranging from £23 to £31 a week. Among those with LSI < 1 disability costs were not significantly different from zero for the least disabled children (quartiles 1–2) but were for the most disabled children in quartiles 3 and 4. Based on the net household income measure, the CV was £46 for families with a child in disability quartile 3, increasing to £80 a week in quartile 4. Similar trends were found for the other income measures.

**Table 6:** Compensating variation by level of disability, living standards and income measure

Disability	Measure of income (mean income difference (95% CI) [N])							
	Net income		Net income + formal care		Discretionary income		Discretionary income + formal care	
	LSI < 1	LSI = 1	LSI < 1	LSI = 1	LSI < 1	LSI = 1	LSI < 1	LSI = 1
All	**22.78 (6.78; 38.79) [2769]**	−16.78 (−74.62; 41.10) [1149]	**30.11 (13.59; 46.63) [2769]**	−12.10 (−70.10; 45.90) [1149]	**24.08 (8.05; 40.11) [2536]**	−32.65 (−94.55; 29.24) [1067]	**31.44 (14.87; 48.00) [2536]**	−27.88 (−89.91; 34.14) [1067]
Quartile 1	13.99 (−15.70; 43.68) [786]	18.11 (−55.16; 91.38) [324]	14.37 (−15.33; 44.08) [786]	18.63 (−54.73; 91.98) [324]	14.04 (−14.98; 43.07) [729]	−10.01 (−64.27; 84.29) [304]	14.46 (−14.59; 43.51) [729]	10.56 (−63.80; 84.92) [304]
Quartile 2	1.44 (−27.22; 30.10) [712]	−7.06 (−133.91; 119.80) [314]	2.50 (−26.23; 31.22) [712]	−4.07 (−131.16; 123.02) [314]	5.01 (−22.61; 32.64) [648]	−5.00 (−135.98; 139.98) [297]	6.17 (−21.53; 33.87) [648]	−1.84 (−132.48; 128.79) [297]
Quartile 3	**45.84 (10.79; 80.89) [491]**	−16.35 (−145.48; 112.78) [214]	**50.71 (14.80; 86.63) [491]**	−13.92 (−143.07; 115.23) [214]	**46.34 (12.31; 80.37) [444]**	−4.60 (−139.98; 130.77) [191]	**51.40 (16.40; 86.40) [444]**	−1.89 (−137.26; 133.48) [191]
Quartile 4	**79.71 (43.90; 115.53)[617]**	9.21 (−88.36; 106.77) [297]	**106.30 (66.54; 146.05) [617]**	21.77 (−76.33; 119.87) [297]	**82.58 (47.94; 117.22) [561]**	1.12 (−100.47; 102.52) [275]	**109.13 (70.07; 148.20) [561]**	13.62 (−88.21; 115.44) [275]

Figures in bold are significantly different from zero at the 5% level

### Receipt of disability benefits and formal care

Mean (SD) disability benefits per week across all children with a disability were £19.19 (£36.12); including formal care the value was £26.55 (£115.61) (Table 7). Weekly disability benefits increased from £4 in the least disabled quartile to £37 in the most disabled quartile. With the addition of the formal care the values were £5 and £71, respectively. There was little difference in weekly disability benefits between living standards groups (Table 8), though when including formal care, benefits were slightly higher among those with the highest living standards.

**Table 7:** Mean benefits and value of formal care by disability level

Disability	Mean (SD) [*N*]	
	Disability benefits	Disability benefits + formal care
All	19.19 (36.12) [3888]	26.55 (115.61) [3888]
Quartile 1	4.04 (15.80) [1143]	4.57 (18.60) [1143]
Quartile 2	7.85 (22.38) [1077]	9.21 (38.10) [1077]
Quartile 3	24.37 (38.78) [726]	29.43 (81.64) [726]
Quartile 4	46.96 (46.11) [942]	70.84 (212.35) [942]

**Table 8:** Mean benefits and value of formal care by disability level and living standards

Disability	Mean (SD) [*N*]			
	Disability benefits		Disability benefits + formal care	
	LSI < 1	LSI = 1	LSI < 1	LSI = 1
All	19.06 (35.77) [2790]	19.51 (37.00) [1098]	27.40 (130.72) [2790]	24.39 (62.49) [1098]
Quartile 1	4.12 (16.11) [830]	3.81 (14.94) [313]	4.65 (18.99) [830]	4.35 (17.56) [313]
Quartile 2	7.24 (21.92) [769]	8.13 (23.53) [308]	8.42 (26.28) [769]	11.17 (57.92) [308]
Quartile 3	24.43 (38.55) [519]	24.22 (39.46) [207]	30.51 (91.85) [519]	26.73 (47.25) [207]
Quartile 4	46.91 (45.45) [672]	47.10 (47.79) [270]	74.83 (244.88) [672]	60.92 (89.56) [270]

### Comparison of CV and receipt of disability benefits and formal care

The findings show differences in disability costs and the value of disability benefits by levels of disability and living standards. Among the highest living standards group, the costs of disability are not significantly different to zero at every disability level, and non-zero benefits are received, irrespective of the income measure used. Among those with LSI < 1, in the least disabled quartiles (quartiles 1 and 2) the trend is the same as for LSI = 1. In disability quartiles 3 and 4 the costs of disability are higher than the benefits received. In quartile 3 the CV is £46–£51 per week and the benefits and formal care value £24–£30 per week; in quartile 4 the CV is £80–£109 per week, and the value of benefits and formal care is £47–£75 per week.

## Conclusion and discussion

In this article we used the notion of compensation variation to estimate the cost of disability among families with a disabled child in the UK. We used a propensity score-matching technique to match families with and without a disabled child with exact matching on living standards.

Our results show that across families with the most disabled children, a compensating variation equal to an extra £56–£79 a week was required to achieve the same living standards as matched families without a disabled child, depending on the measure of income used. These figures varied by living standards and disability: in the higher living standards group the CV was not significantly different to zero with any income measure or at any disability level. Among families with a disabled child with lower living standards, disability costs were £23–£31 a week across all families with a disabled child and not significantly different from zero for the least disabled children but were for the most disabled children in quartiles 3 and 4. In the latter groups, the costs of disability were substantial, with a CV of £46–£109 per week depending on the income measure used. In these families the value of the disability benefits received was lower than the compensating variation. Given the appreciably lower family income among families in the lower living standard groups shown by our data, this suggests the costs of child disability are relatively high among relatively low income families with children with more severe disabilities. Our conclusion is that given the discrepancies between the costs of child disability and receipt of benefits in these groups, this suggests that child disability benefits should be targeted more carefully at low income families with more severely disabled children.^12^

Our study has several strengths. To our knowledge it is the first to quantify the cost of child disability in the UK using a propensity score matching approach. We employed a large UK-wide representative data set, which ensured that our analyses were powered to detect differences, especially given the low prevalence of child disability. Moreover, we had rich data on a number of family financial indicators such as income, benefits, expenditure, and savings.

There are several limitations. First, in terms of the disability measures, due to the data collected in the FRS, our disability indices account for the impact of each condition relative to one other, but not for the severity of each condition. For example, there may be considerable variation in the extent of ‘mobility problems’ but we are not able to account for the impact of these in our disability index. Second, we have only included families with at most one disabled child because of difficulties in disaggregating the impact of having multiple disabled children in a family.^13^ Third, our living standard index measures the quantity of the items families can and cannot afford, but not the quality of those items; this perhaps explains the grouping of families at the highest level of living standards in our data. Fourth, we have not disaggregated disability costs by type of disability, for example related to mental versus physical health problems, which would be a potentially useful extension of this work. Fifth, the aim of this article was to estimate the cost of child disability borne directly by the families, which is a part of the total cost of child disability to the society. To access the total cost of child disability from the societal point of view, one ought to add the costs borne by the formal sector (for instance, healthcare, social security, and education).

### Electronic supplementary material

Below is the link to the electronic supplementary material.

Supplementary material 1 (DOCX 88 kb)

## Appendix

See Tables 9, 10, 11, 12 and 13.

**Table 9:** Covariates used to construct the Disability Index

Covariate	Definition
Child characteristics	
Child age	Age in years at last birthday
Child gender	Female
Family characteristics	
Government region	London; South East; Wales, Scotland, Northern Ireland; Rest of England
House value	Council tax band
Homeowner	Homeowner outright or with mortgage
Social tenant	Rents home from council or housing association
Savings	Estimates value of accounts/investments
Number of children	
Schooling	Number of years’ schooling beyond 18
Areas affected by disability	
Mobility	Problems with moving about
Lifting	Problems with lifting, carrying or moving objects
Dexterity	Problems with using hands to carry out everyday tasks
Incontinence	Problems with bladder and bowel control
Communication	Problems with speech, hearing or eyesight
Memory	Problems with memory or ability to concentrate, learn or understand
Danger	Problems with recognising when you are in physical danger
Co-ordination	Problems with physical co-ordination and balance
Other	Other health problem or disability

**Table 10:** Questions employed to construct the Living Standard Index

Living standard questions
1. Do you and your family have a holiday away from home for at least 1 week a year, whilst not staying with relatives at their home?
2. Do you have enough money to keep your house in a decent state of decoration?
3. Do you have household contents insurance?
4. Do you make regular savings of £10 a month or more for rainy days or retirement?
5. Do you replace any worn out furniture?
6. Do you replace or repair major electrical goods such as a refrigerator or a washing machine when broken?
7. Do you have a small amount of money to spend each week on yourself (not on your family)?
8. Does your child/do your children have a family holiday away from home for at least 1 week a year?
9. Does your child/do your children have leisure equipment such as sports equipment or a bicycle?
10. Does your child/do your children have celebrations on special occasions such as birthdays, Christmas or other religious festivals?
11. Does your child/do your children do a hobby or leisure activity?
12. Does your child/do your children have friends around for tea or a snack once a fortnight?

**Table 11:** Estimation results: Disability Index

Covariate	Logit coefficients	95% CI
Area affected (as in Table 2)		
Child age	0.008	−0.01; 0.03
Child gender	−0.178	−0.35; −0.01
Government region		
London	−0.133	−0.45; 0.18
South East	0.149	−0.10; 0.40
Wales, Scotland, Northern Ireland and Rest of England (omitted)	0.199	−0.01; 0.41
House value		
Band A (omitted)		
Band B	−0.188	−0.41; 0.04
Band C	−0.268	−0.51; −0.02
Band D	−0.015	−0.30; 0.27
Band E	−0.270	−0.64; 0.10
Band F	−0.460	−0.95; 0.03
Band G	−0.359	−0.96; 0.24
Band H	−2.391	−4.53; −0.25
Homeowner	−0.115	−0.38; 0.15
Social tenant	−0.068	−0.32; 0.19
Savings		
Less than 1500 (omitted)		
From 1500 up to 3000	−0.135	−0.46; 0.20
From 3000 up to 8000	0.186	−0.13; 0.50
From 8000 up to 20,000	−0.173	−0.58; 0.23
From 20,000 up to 25,000	−0.023	−0.68; 0.63
From 25,000 up to 30,000	0.378	−0.43; 1.18
From 30,000 up to 35,000	0.623	−0.21; 1.45
From 35,000 up to 40,000	0.484	−0.31; 1.28
Over 40,000	0.193	−0.25; 0.63
N/A	−0.506	−1.15; 0.13
Number of children	−0.005	−0.08; 0.07
Schooling	−0.019	−0.07; 0.03
Year		
2004/05 (omitted)		
2005/06	−0.251	−0.55; 0.04
2006/07	−0.524	−0.84; −0.21
2007/08	−0.150	−0.45; 0.15
2008/09	−0.199	−0.50; 0.11
2009/10	−0.234	−0.53; 0.07
2010/11	−0.116	−0.41; 0.18
2011/12	−0.029	−0.34; 0.29
Constant	−1.765	−2.20; −1.33
*N*	3952

**Table 12:** Equalised income, square root of family size, Burniaux et al. [4]

Disability	Mean income difference (95% CI) [*N*]		
	All LSI	LSI < 1	LSI = 1
All	**12.01 (2.70; 21.32) [3922]**	**9.11 (1.74; 16.49) [2772]**	19.01 (−7.31; 45.34) [1,150]
Quartile 1	2.74 (−11.58; 17.07) [1105]	7.00 (−6.68; 20.68) [781]	−7.51 (−43.70: 28.67) [324]
Quartile 2	−11.04 (−37.45; 15.36) [1028]	−4.18 (−18.63; 10.26) [712]	−26.50 (−106.21; 53.21) [316]
Quartile 3	16.17 (−5.02; 37.36) [711]	**20.07 (2.14; 37.99) [498]**	7.05 (−50.18; 64.30) [213]
Quartile 4	**27.87 (9.44; 46.31) [932]**	**35.83 (17.26; 54.41) [635]**	10.85 (−31.33; 53.04) [297]

**Table 13:** Equalised income, exact matching on the number of adults

Disability	Mean income difference (95% CI) [*N*]		
	All LSI	LSI < 1	LSI = 1
All	11.96 (−7.59; 31.52) [3759]	8.16 (−6.89; 23.22) [2607]	20.57 (−30.55; 71.70) [1152/1153]
Quartile 1	4.66 (−24.59; 33.92) [1066]	2.89 (−25.81; 31.60) [742]	8.70 (−61.82; 79.23) [324/324]
Quartile 2	−7.58 (−62.97; 47.80) [986]	0.49 (−28.65; 29.63) [670]	−24.69 (−186.53; 137.15) [316]
Quartile 3	24.63 (−26.90; 76.17) [683]	**42.82 (6.39; 79.26) [470]**	−15.51 (−160.51; 129.48) [213]
Quartile 4	**58.00 (17.79; 98.20) [890]**	**73.92 (36.55; 111.30) [593]**	26.19 (−68.68; 121.08) [297]

Figures in bold are significantly different from zero at the 5% level

## Footnotes

1
https://www.gov.uk/disability-living-allowance-children/rates.

2 The age for DLA for children has been changed since 1997/1998–16.

3 Following Hancock et al. [17], the compensation variation for a family with a disabled child on income Y_0_ is also the equivalent variation (with an opposite sign, i.e. an income loss that is welfare-equivalent to the onset of disability) for a family without a disabled child initially on income *Y*_0_ + CV′.

4 A comprehensive review of the relationship between standards of living and CV is given in Zaidi and Burchardt [38].

5 The responders were given the option of saying that an item did not apply for them. For instance, a potential ‘necessity’ to afford sports equipment or a bicycle is related to the age of a child; the same is related to having a hobby or leisure activity.

6 Participants are defined as being able to afford an item if they said they owned it or they said they could afford it but did not want it. With this specification we allow for heterogeneity in preferences to explain non-consumption among families with and without disabled children rather than excluding items that could be irrelevant because of disability, i.e. having a bicycle or sport equipment.

7 We did not include the NHS cost because the costs of healthcare are not covered by disability benefits and do not contribute to the family income.

8 In line with previous work [26, 38] we use the set of household characteristics to adjust for household composition rather than use an arbitrary equivalence scale. Nevertheless, in the Appendix (Tables 12 and 13) we have also provided the results with equalised income using two different methods.

9 We have also estimated the main model with the calliper size of one-fifth and on third of the standard deviation range of the estimated propensity score, the results are very similar and are available upon request.

10 The estimate coefficients of the logit model are summarised in Appendix Table 3.

11 Statistical tests are available in the Supplementary Materials.

12 Our findings show that the CVs in families with the highest living standards are not significantly different from zero, but this does not suggest there should be a reallocation of benefits away from these families towards those with lower living standards; on the contrary, our findings suggest increased support for low income families of disabled children is warranted, leaving other families with disabled children unaffected. Also, it is worth noting that these findings do not account for incentives, problems and potential poverty traps induced by them.

13 The number of families with two or more disable children were quite small (about 2%) to use our methodology in a robust way.
