# Supplementary material for: Using compensating variation to measure the costs of child disability in the UK
Source: Eur J Health Econ. 2017 Apr 13;19(3):419–33. doi: 10.1007/s10198-017-0893-7 (PMC5978910; doi:10.1007/s10198-017-0893-7)
Supplement: Supplementary file 1 — Supplementary material 1 (DOCX 88 kb) [file 10198_2017_893_MOESM1_ESM.docx]

**Supplementary Materials**

| Table S1. Areas affected by disability | | | | | | |
| --- | --- | --- | --- | --- | --- | --- |
|  |  | Total number of disabled children in family | | | | |
| Area affected by disability | Number of children having a disability | 1 | 2 | 3 | 4 | 5 |
| Mobility | 1 | 1,220 | 184 | 24 | 5 | 3 |
|  | 2 | 0 | 148 | 20 | 8 | 0 |
|  | 3 | 0 | 0 | 30 | 0 | 0 |
| Lifting | 1 | 557 | 89 | 13 | 4 | 2 |
|  | 2 | 0 | 44 | 4 | 0 | 0 |
|  | 3 | 0 | 0 | 18 | 0 | 0 |
| Dexterity | 1 | 694 | 108 | 15 | 3 | 1 |
|  | 2 | 0 | 58 | 8 | 4 | 2 |
|  | 3 | 0 | 0 | 18 | 3 | 0 |
| Incontinence | 1 | 680 | 131 | 34 | 5 | 2 |
|  | 2 | 0 | 50 | 8 | 0 | 2 |
|  | 3 | 0 | 0 | 12 | 0 | 0 |
| Communication | 1 | 1,534 | 227 | 33 | 6 | 1 |
|  | 2 | 0 | 212 | 46 | 6 | 0 |
|  | 3 | 0 | 0 | 30 | 0 | 0 |
| Memory | 1 | 1,629 | 236 | 34 | 3 | 1 |
|  | 2 | 0 | 240 | 42 | 10 | 0 |
|  | 3 | 0 | 0 | 33 | 3 | 6 |
|  | 4 | 0 | 0 | 0 | 4 | 0 |
|  | 5 | 0 | 0 | 0 | 0 | 5 |
| Appreciation of danger | 1 | 1,150 | 178 | 29 | 3 | 2 |
|  | 2 | 0 | 144 | 22 | 10 | 0 |
|  | 3 | 0 | 0 | 24 | 3 | 3 |
|  | 4 | 0 | 0 | 0 | 0 | 0 |
|  | 5 | 0 | 0 | 0 | 0 | 5 |
| Co-ordination | 1 | 1,020 | 162 | 26 | 6 | 2 |
|  | 2 | 0 | 196 | 20 | 4 | 0 |
|  | 3 | 0 | 0 | 24 | 0 | 3 |
| Other | 1 | 1,865 | 250 | 34 | 2 | 2 |
|  | 2 | 0 | 368 | 50 | 4 | 2 |
|  | 3 | 0 | 0 | 54 | 9 | 3 |
|  | 4 | 0 | 0 | 0 | 12 | 0 |
| Total disabled children |  | 4,320 | 1,308 | 292 | 43 | 20 |

| Table S2.Balance after matching for Net^[[1]](#footnote-1)^ Income and Disability level 1 | | | | | | | |
| --- | --- | --- | --- | --- | --- | --- | --- |
|  | Unmatched (U) | Mean | | %reduct | | t-test | |
| Variable | Matched (M) | Treated | Control | %bias | \|bias\| | t | p>\|t\| |
| Age | U | 8.43 | 7.34 | 24.00 | 8.18 | 0.00 |  |
|  | M | 8.44 | 8.21 | 5.00 | 79.20 | 1.21 | 0.23 |
| Sex | U | 0.43 | 0.50 | -13.80 | -4.81 | 0.00 |  |
|  | M | 0.43 | 0.44 | -1.40 | 89.50 | -0.34 | 0.73 |
| London | U | 0.08 | 0.10 | -7.90 | -2.62 | 0.01 |  |
|  | M | 0.08 | 0.08 | -0.30 | 96.00 | -0.08 | 0.94 |
| South England | U | 0.10 | 0.12 | -6.10 | -2.07 | 0.04 |  |
|  | M | 0.10 | 0.09 | 2.60 | 57.30 | 0.65 | 0.52 |
| Wales/Scotland/Northern Ireland | U | 0.28 | 0.28 | 1.50 | 0.51 | 0.61 |  |
|  | M | 0.28 | 0.30 | -3.80 | -162.00 | -0.89 | 0.38 |
| Family composition | U | 1.63 | 1.75 | -28.20 | -10.51 | 0.00 |  |
|  | M | 1.66 | 1.66 | 0.00 | 100.00 | 0.00 | 1.00 |
| Disabled adult | U | 0.39 | 0.15 | 56.00 | 23.38 | 0.00 |  |
|  | M | 0.35 | 0.35 | 0.20 | 99.60 | 0.04 | 0.96 |
| Schooling | U | 0.68 | 1.10 | -21.20 | -6.87 | 0.00 |  |
|  | M | 0.71 | 0.67 | 1.70 | 91.90 | 0.43 | 0.67 |
| Number of children | U | 2.07 | 2.19 | -12.20 | -4.20 | 0.00 |  |
|  | M | 2.09 | 2.06 | 3.10 | 74.40 | 0.78 | 0.44 |
| Unemployed | U | 0.04 | 0.04 | 3.90 | 1.42 | 0.16 |  |
|  | M | 0.04 | 0.04 | 0.00 | 100.00 | 0.00 | 1.00 |
| Out of Labour Force | U | 0.36 | 0.25 | 23.90 | 8.84 | 0.00 |  |
|  | M | 0.33 | 0.34 | -2.60 | 89.20 | -0.58 | 0.56 |
| Savings <1,500 | U | 0.66 | 0.53 | 25.00 | 8.57 | 0.00 |  |
|  | M | 0.64 | 0.65 | -1.80 | 92.60 | -0.44 | 0.66 |
| Savings 1,500-20,000 | U | 0.18 | 0.26 | -20.70 | -6.81 | 0.00 |  |
|  | M | 0.19 | 0.18 | 2.90 | 86.20 | 0.71 | 0.48 |
| Savings >20,000 | U | 0.08 | 0.13 | -15.10 | -4.84 | 0.00 |  |
|  | M | 0.09 | 0.08 | 2.40 | 84.20 | 0.61 | 0.54 |
| Savings not available | U | 0.02 | 0.03 | -10.30 | -3.16 | 0.00 |  |
|  | M | 0.02 | 0.02 | 0.00 | 100.00 | 0.00 | 1.00 |

| Table S3.Balance after matching for Net Income and Disability level 2 | | | | | | | |
| --- | --- | --- | --- | --- | --- | --- | --- |
|  | Unmatched (U) | Mean | | %reduct | | t-test | |
| Variable | Matched (M) | Treated | Control | %bias | \|bias\| | t | p>\|t\| |
|  |  |  |  |  |  |  |  |
| Age | U | 9.04 | 7.34 | 38.3 | 12.36 | 0.00 |  |
|  | M | 9.03 | 9.03 | 0.1 | 99.70 | 0.03 | 0.98 |
| Sex | U | 0.38 | 0.50 | -23.3 | -7.83 | 0.00 |  |
|  | M | 0.40 | 0.39 | 2.4 | 89.80 | 0.54 | 0.59 |
| London | U | 0.09 | 0.10 | -4.8 | -1.59 | 0.11 |  |
|  | M | 0.08 | 0.07 | 1.3 | 72.60 | 0.34 | 0.74 |
| South England | U | 0.12 | 0.12 | -0.3 | -0.09 | 0.93 |  |
|  | M | 0.12 | 0.11 | 2.1 | -680.20 | 0.48 | 0.63 |
| Wales/Scotland/Northern Ireland | U | 0.25 | 0.28 | -5.5 | -1.85 | 0.07 |  |
|  | M | 0.26 | 0.29 | -6.9 | -24.60 | -1.54 | 0.12 |
| Family composition | U | 1.59 | 1.75 | -35.7 | -13.01 | 0.00 |  |
|  | M | 1.62 | 1.62 | 0.8 | 97.60 | 0.18 | 0.86 |
| Disabled adult | U | 0.35 | 0.15 | 47.6 | 19.03 | 0.00 |  |
|  | M | 0.31 | 0.31 | -0.7 | 98.50 | -0.14 | 0.89 |
| Schooling | U | 0.64 | 1.10 | -24.4 | -7.36 | 0.00 |  |
|  | M | 0.66 | 0.56 | 4.8 | 80.20 | 1.34 | 0.18 |
| Number of children | U | 2.12 | 2.19 | -6.8 | -2.32 | 0.02 |  |
|  | M | 2.13 | 2.17 | -3.2 | 52.90 | -0.73 | 0.47 |
| Unemployed | U | 0.05 | 0.04 | 6.8 | 2.50 | 0.01 |  |
|  | M | 0.05 | 0.04 | 4.9 | 28.10 | 1.10 | 0.27 |
| Out of Labour Force | U | 0.38 | 0.25 | 29.9 | 10.81 | 0.00 |  |
|  | M | 0.35 | 0.33 | 4.2 | 85.80 | 0.93 | 0.35 |
| Savings <1,500 | U | 0.65 | 0.53 | 23.3 | 7.76 | 0.00 |  |
|  | M | 0.62 | 0.63 | -2.6 | 88.90 | -0.59 | 0.55 |
| Savings 1,500-20,000 | U | 0.19 | 0.26 | -17.6 | -5.68 | 0.00 |  |
|  | M | 0.21 | 0.20 | 1.4 | 92.00 | 0.33 | 0.74 |
| Savings >20,000 | U | 0.09 | 0.13 | -12.7 | -4.00 | 0.00 |  |
|  | M | 0.10 | 0.09 | 1.3 | 90.00 | 0.30 | 0.77 |
| Savings not available | U | 0.02 | 0.03 | -5.4 | -1.70 | 0.09 |  |
|  | M | 0.02 | 0.02 | 2.4 | 55.50 | 0.60 | 0.55 |

| Table S4.Balance after matching for Net Income and Disability level 3 | | | | | | | |
| --- | --- | --- | --- | --- | --- | --- | --- |
|  | Unmatched (U) | Mean | | %reduct | | t-test | |
| Variable | Matched (M) | Treated | Control | %bias | \|bias\| | t | p>\|t\| |
|  |  |  |  |  |  |  |  |
| Age | U | 9.50 | 7.34 | 49.30 | 13.09 | 0.00 |  |
|  | M | 9.30 | 8.99 | 7.10 | 85.60 | 1.43 | 0.15 |
| Sex | U | 0.35 | 0.50 | -29.60 | -8.24 | 0.00 |  |
|  | M | 0.36 | 0.35 | 2.60 | 91.20 | 0.50 | 0.62 |
| London | U | 0.10 | 0.10 | -2.50 | -0.69 | 0.49 |  |
|  | M | 0.10 | 0.10 | -1.90 | 23.40 | -0.36 | 0.72 |
| South England | U | 0.12 | 0.12 | 0.60 | 0.17 | 0.87 |  |
|  | M | 0.12 | 0.11 | 1.80 | -197.00 | 0.34 | 0.74 |
| Wales/Scotland/Northern Ireland | U | 0.28 | 0.28 | 1.60 | 0.45 | 0.66 |  |
|  | M | 0.29 | 0.32 | -6.00 | -285.20 | -1.10 | 0.27 |
| Family composition | U | 1.62 | 1.75 | -30.30 | -9.19 | 0.00 |  |
|  | M | 1.66 | 1.65 | 0.60 | 98.00 | 0.11 | 0.91 |
| Disabled adult | U | 0.32 | 0.15 | 40.40 | 13.33 | 0.00 |  |
|  | M | 0.28 | 0.29 | -2.40 | 94.10 | -0.41 | 0.68 |
| Schooling | U | 0.65 | 1.10 | -22.90 | -5.98 | 0.00 |  |
|  | M | 0.69 | 0.71 | -1.10 | 95.00 | -0.24 | 0.81 |
| Number of children | U | 2.12 | 2.19 | -6.80 | -1.93 | 0.05 |  |
|  | M | 2.12 | 2.09 | 3.70 | 45.60 | 0.74 | 0.46 |
| Unemployed | U | 0.04 | 0.04 | 4.00 | 1.21 | 0.23 |  |
|  | M | 0.04 | 0.04 | 1.50 | 63.60 | 0.28 | 0.78 |
| Out of Labour Force | U | 0.42 | 0.25 | 37.20 | 11.35 | 0.00 |  |
|  | M | 0.38 | 0.39 | -1.50 | 95.90 | -0.27 | 0.78 |
| Savings <1,500 | U | 0.66 | 0.53 | 26.10 | 7.23 | 0.00 |  |
|  | M | 0.63 | 0.66 | -5.20 | 79.90 | -1.00 | 0.32 |
| Savings 1,500-20,000 | U | 0.18 | 0.26 | -20.20 | -5.40 | 0.00 |  |
|  | M | 0.20 | 0.19 | 4.10 | 79.60 | 0.81 | 0.42 |
| Savings >20,000 | U | 0.08 | 0.13 | -13.50 | -3.56 | 0.00 |  |
|  | M | 0.10 | 0.10 | -2.30 | 82.90 | -0.44 | 0.66 |
| Savings not available | U | 0.01 | 0.03 | -12.30 | -2.98 | 0.00 |  |
|  | M | 0.01 | 0.01 | 0.00 | 100.00 | 0.00 | 1.00 |

| Table S5.Balance after matching for Net Income and Disability level 4 | | | | | | | |
| --- | --- | --- | --- | --- | --- | --- | --- |
|  | Unmatched (U) | Mean | | %reduct | | t-test | |
| Variable | Matched (M) | Treated | Control | %bias | \|bias\| | t | p>\|t\| |
|  |  |  |  |  |  |  |  |
| Age | U | 9.11 | 7.34 | 41.70 | 12.38 | 0.00 |  |
|  | M | 9.12 | 9.15 | -0.90 | 97.90 | -0.20 | 0.84 |
| Sex | U | 0.29 | 0.50 | -43.60 | -13.62 | 0.00 |  |
|  | M | 0.31 | 0.32 | -1.40 | 96.80 | -0.30 | 0.76 |
| London | U | 0.09 | 0.10 | -4.80 | -1.53 | 0.13 |  |
|  | M | 0.08 | 0.10 | -6.70 | -38.30 | -1.47 | 0.14 |
| South England | U | 0.13 | 0.12 | 4.60 | 1.55 | 0.12 |  |
|  | M | 0.13 | 0.13 | 1.00 | 78.40 | 0.21 | 0.83 |
| Wales/Scotland/Northern Ireland | U | 0.25 | 0.28 | -6.40 | -2.07 | 0.04 |  |
|  | M | 0.25 | 0.25 | 1.00 | 84.50 | 0.22 | 0.83 |
| Family composition | U | 1.64 | 1.75 | -25.20 | -8.72 | 0.00 |  |
|  | M | 1.67 | 1.72 | -9.40 | 62.90 | -1.98 | 0.05 |
| Disabled adult | U | 0.33 | 0.15 | 44.20 | 16.91 | 0.00 |  |
|  | M | 0.27 | 0.28 | -1.60 | 96.40 | -0.31 | 0.75 |
| Schooling | U | 0.73 | 1.10 | -18.50 | -5.65 | 0.00 |  |
|  | M | 0.78 | 0.80 | -1.10 | 93.90 | -0.24 | 0.81 |
| Number of children | U | 2.12 | 2.19 | -6.90 | -2.24 | 0.03 |  |
|  | M | 2.15 | 2.10 | 5.60 | 18.30 | 1.25 | 0.21 |
| Unemployed | U | 0.04 | 0.04 | 2.90 | 0.99 | 0.32 |  |
|  | M | 0.04 | 0.03 | 5.70 | -95.80 | 1.22 | 0.22 |
| Out of Labour Force | U | 0.45 | 0.25 | 43.30 | 15.25 | 0.00 |  |
|  | M | 0.39 | 0.40 | -1.90 | 95.70 | -0.38 | 0.70 |
| Savings <1,500 | U | 0.64 | 0.53 | 22.00 | 7.06 | 0.00 |  |
|  | M | 0.60 | 0.59 | 2.50 | 88.80 | 0.52 | 0.60 |
| Savings 1,500-20,000 | U | 0.20 | 0.26 | -13.90 | -4.36 | 0.00 |  |
|  | M | 0.23 | 0.26 | -8.60 | 38.30 | -1.80 | 0.07 |
| Savings >20,000 | U | 0.09 | 0.13 | -11.40 | -3.50 | 0.00 |  |
|  | M | 0.11 | 0.09 | 4.60 | 59.90 | 1.02 | 0.31 |
| Savings not available | U | 0.02 | 0.03 | -9.90 | -2.83 | 0.01 |  |
|  | M | 0.02 | 0.02 | -2.10 | 78.30 | -0.51 | 0.61 |

| Table S6.Balance after matching for Net Income and All Disability levels | | | | | | | |
| --- | --- | --- | --- | --- | --- | --- | --- |
|  | Unmatched (U) | Mean | | %reduct | | t-test | |
| Variable | Matched (M) | Treated | Control | %bias | \|bias\| | t | p>\|t\| |
|  |  |  |  |  |  |  |  |
| Age | U | 8.97 | 7.34 | 36.80 | 22.38 | 0.00 |  |
|  | M | 8.96 | 8.57 | 8.80 | 76.00 | 4.08 | 0.00 |
| Sex | U | 0.37 | 0.50 | -26.60 | -16.76 | 0.00 |  |
|  | M | 0.37 | 0.40 | -5.00 | 81.20 | -2.23 | 0.03 |
| London | U | 0.09 | 0.10 | -5.20 | -3.25 | 0.00 |  |
|  | M | 0.08 | 0.09 | -2.40 | 53.50 | -1.12 | 0.26 |
| South England | U | 0.12 | 0.12 | -0.50 | -0.31 | 0.76 |  |
|  | M | 0.12 | 0.11 | 1.60 | -231.70 | 0.71 | 0.48 |
| Wales/Scotland/Northern Ireland | U | 0.27 | 0.28 | -2.40 | -1.50 | 0.13 |  |
|  | M | 0.27 | 0.27 | 0.50 | 80.50 | 0.20 | 0.84 |
| Family composition | U | 1.62 | 1.75 | -29.90 | -20.29 | 0.00 |  |
|  | M | 1.64 | 1.66 | -4.20 | 85.90 | -1.80 | 0.07 |
| Disabled adult | U | 0.35 | 0.15 | 47.80 | 35.54 | 0.00 |  |
|  | M | 0.33 | 0.29 | 9.30 | 80.60 | 3.75 | 0.00 |
| Schooling | U | 0.67 | 1.10 | -21.70 | -12.79 | 0.00 |  |
|  | M | 0.70 | 0.71 | -0.60 | 97.40 | -0.27 | 0.79 |
| Number of children | U | 2.11 | 2.19 | -8.40 | -5.32 | 0.00 |  |
|  | M | 2.12 | 2.10 | 2.00 | 76.30 | 0.90 | 0.37 |
| Unemployed | U | 0.04 | 0.04 | 4.50 | 3.02 | 0.00 |  |
|  | M | 0.04 | 0.04 | 0.50 | 88.30 | 0.23 | 0.82 |
| Out of Labour Force | U | 0.40 | 0.25 | 32.90 | 22.39 | 0.00 |  |
|  | M | 0.37 | 0.35 | 5.90 | 82.20 | 2.49 | 0.01 |
| Savings <1,500 | U | 0.65 | 0.53 | 24.00 | 15.06 | 0.00 |  |
|  | M | 0.63 | 0.64 | -2.10 | 91.10 | -0.96 | 0.34 |
| Savings 1,500-20,000 | U | 0.19 | 0.26 | -18.00 | -10.98 | 0.00 |  |
|  | M | 0.20 | 0.20 | 0.50 | 97.30 | 0.23 | 0.82 |
| Savings >20,000 | U | 0.09 | 0.13 | -13.20 | -7.88 | 0.00 |  |
|  | M | 0.09 | 0.09 | 1.80 | 86.10 | 0.87 | 0.39 |
| Savings not available | U | 0.02 | 0.03 | -9.20 | -5.24 | 0.00 |  |
|  | M | 0.02 | 0.02 | -0.50 | 94.60 | -0.24 | 0.81 |

**Figure S1**


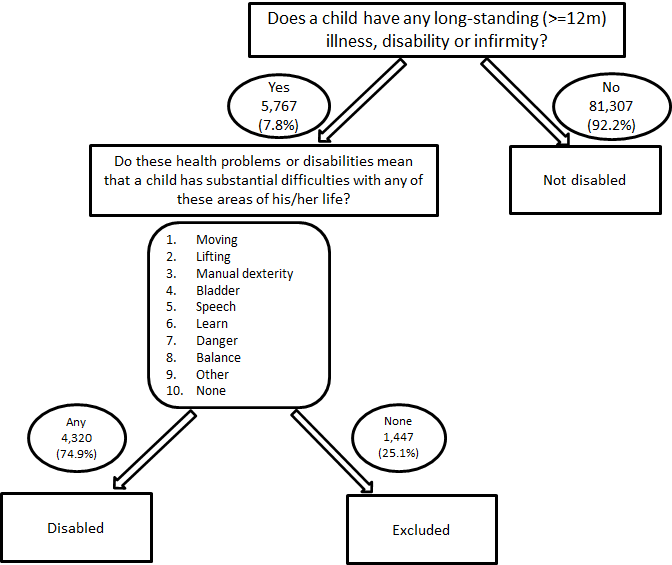


1. Findings for other income measures were very similar. [↑](#footnote-ref-1)
